# Electroconvulsive Therapy and Hyperventilation: A Narrative Review

**DOI:** 10.3390/life15091368

**Published:** 2025-08-28

**Authors:** Joanna Smolarczyk, Patrycja Piłat, Jordi Blanch, Aleksandra Cetnarowska, Paweł Dębski, Aurora Torrent, Iolanda Batalla, Magdalena Piegza

**Affiliations:** 1 Department of Psychoprophylaxis, Medical University of Silesia, 40-055 Katowice, Poland; 2 Department of Psychiatry, Faculty of Medical Sciences in Zabrze, Medical University of Silesia, 40-055 Katowice, Poland; 3 Department of Psychiatry, University of Barcelona, Villarroel 140, 08036 Barcelona, Spain; 4 Department of Psychiatry, Hospital Universitari de Santa Maria, 25198 Lleida, Spain

**Keywords:** electroconvulsive therapy, hyperventilation, treatment

## Abstract

Electroconvulsive therapy (ECT) is a non-pharmacological biological treatment method used to treat major depression, bipolar disorder, schizophrenia, catatonia, and some other psychiatric conditions. Despite its high effectiveness, it is often used when other methods, such as pharmacotherapy and psychotherapy, fail to improve treatment outcomes. The refinement of this particular therapy may increase the popularity of this method, and among the currently studied therapy modifiers is protocolised hyperventilation. Hyperventilation is implemented to improve ventilation and gas exchange, reduce shortness of breath, improve blood oxygenation, and prevent hypoxia. Research suggests that hyperventilation during ECT may prolong the duration of epileptic seizures, potentially enhancing the effectiveness of the therapy. However, research on hyperventilation during ECT still poses many questions regarding its benefits and side effects. Innovative studies on ECT with concomitant hyperventilation focus on monitoring parameters such as CO_2_, EEG, and cardiovascular responses. Current research directions worth exploring also include the utilisation of modern ECT devices or determining the neurotrophin concentration to better understand the mechanism of action at the neurochemical level. The personalization of therapy, including adjustment of ECT parameters to patients’ specific symptoms, can reduce the risk of failure and increase effectiveness.

## 1. Introduction

Electroconvulsive therapy (ECT) is a non-pharmacological biological treatment method that is most often used to treat patients with severe depression or bipolar disorder. It is also used in the treatment of schizophrenia and catatonia, as well as in individual cases of Parkinson’s disease, epilepsy, and dystonia. ECT is usually used when other therapy methods, such as pharmacotherapy and psychotherapy, could not have caused any improvement. ECT is also used in people who require a timely, immediate response to treatment due to the severity of their condition, such as suicide risk [1,2,3]. Despite its high effectiveness, the main limitation of ECT is the significant rate of disease recurrence after treatment. Hence, several courses and supportive therapies are recommended [4]. The advantage of ECT is its very high effectiveness, up to 60–70% [3]. However, the efficiency itself is not related to the popularity of this method. ECT is controversial due to the use of voltage in this therapy, which is associated with a risk of adverse events. It is seemingly easier for patients to choose known and cheaper methods, such as treatment with pharmacological agents. Moreover, there are many theories about ECT, but the mechanism of the therapeutic effect of electrical current-induced seizures has not been fully understood yet. Current evidence suggests that generalised seizures have a potent impact on diencephalic structures, with the hypothalamus playing a pivotal role in alleviating the symptoms of depression [4]. In addition, generalised seizures cause neurotrophic changes in the hippocampus [5].

**Figure 1:**
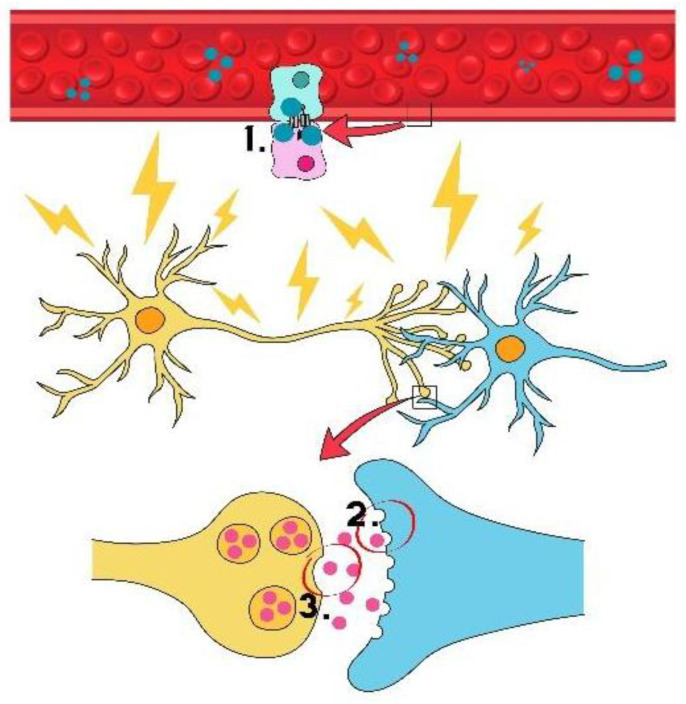
Neuroplasticity associated with ECT stimulation: 1—increasing the permeability of the blood–brain barrier; 2—increasing the activity of receptors and their affinity to neurotransmitters; 3—stimulating neurotransmitter release in the central nervous system (CNS). The figure was created using Canva Pro.

Both animal and human studies suggest a potential for neuroplasticity associated with ECT stimulation, including neurogenesis, synaptogenesis, angiogenesis, or gliogenesis [6]. Such changes occur by prompting the release of neurotransmitters in the central nervous system (CNS), stimulating the disturbed hypothalamic–pituitary axis, increasing the permeability of the blood-brain barrier, and elevating the activity of receptors and their affinity for transmitters (Figure 1) [7]. However, as with any therapy, adverse effects may occur. The most common undesirable effects of ECT are memory disorders, especially short-term memory loss concerning recent events, which cease within the next few days or weeks, even though in some patients, they may persist for several months [8]. Rarely, more aggravated complications may occur, such as postictal agitation, cardiovascular disorders, prolonged epileptic seizures, and status epilepticus [9]. A 2021 study conducted at a psychiatric hospital in northern Bavaria, Germany, analysed 157 patients who underwent 3106 ECT sessions over three years and found a low incidence of severe, potentially life-threatening adverse events requiring intervention, amounting to 0.097%. Considering the available evidence, we can conclude that ECT is a safe method [10].

Currently, ECT is performed under general anaesthesia, with the use of neuromuscular blocking drugs, and supervised by an anaesthetist. Despite safety concerns, the method is indicated particularly for patients with severe mental disorders during pregnancy, in cases of drug-resistant diseases, extremely high risk of suicide, psychotic agitation, and extreme physical weakness caused by malnutrition or dehydration [11]. Moreover, it should be noted that ECT sessions, regardless of the indications, are not used as a singular or exclusive method but are combined with continued pharmacotherapy [3]. Opportunities are being sought to modify the treatment to maximise the potential of ECT therapy. An increase in the effectiveness of ECT was recorded when the patient was hyperventilated before undergoing the procedure. A correlation has been noted between a decrease in the arterial blood carbon dioxide partial pressure and an increase in the duration of the singular ECT-induced seizure, which potentially translates to a lower electric current energy demanded for ECT treatment [12].

### 1.1. Aim

A review of the current literature in terms of assessing the usefulness of hyperventilation in electroconvulsive therapy.

### 1.2. Materials and Methods

The method used in preparing this study was a narrative review. A search in the PubMed database was used. The following search term combinations were used: “electroconvulsive therapy and innovations”, “electroconvulsive therapy and hyperventilation”, “electroconvulsive therapy and new methods treatment”, and “future electroconvulsive therapy”.

A database survey revealed 1542 items. Automatic article selection tools were used. Articles published since 2000 and with full text were used as inclusion criteria. Reports presented only as abstracts and in the form of books and documents other than articles were excluded.

Such solutions enabled access to articles presenting current research, as well as describing full methodological procedures. Using the inclusion and exclusion parameters, 574 articles were selected for review. The usefulness of the articles was then assessed by two independent scholars, who qualified 92 articles for in-depth analysis. Due to further exclusions, 40 bibliographic sources were used in the preparation of this review.

**Figure 2:**
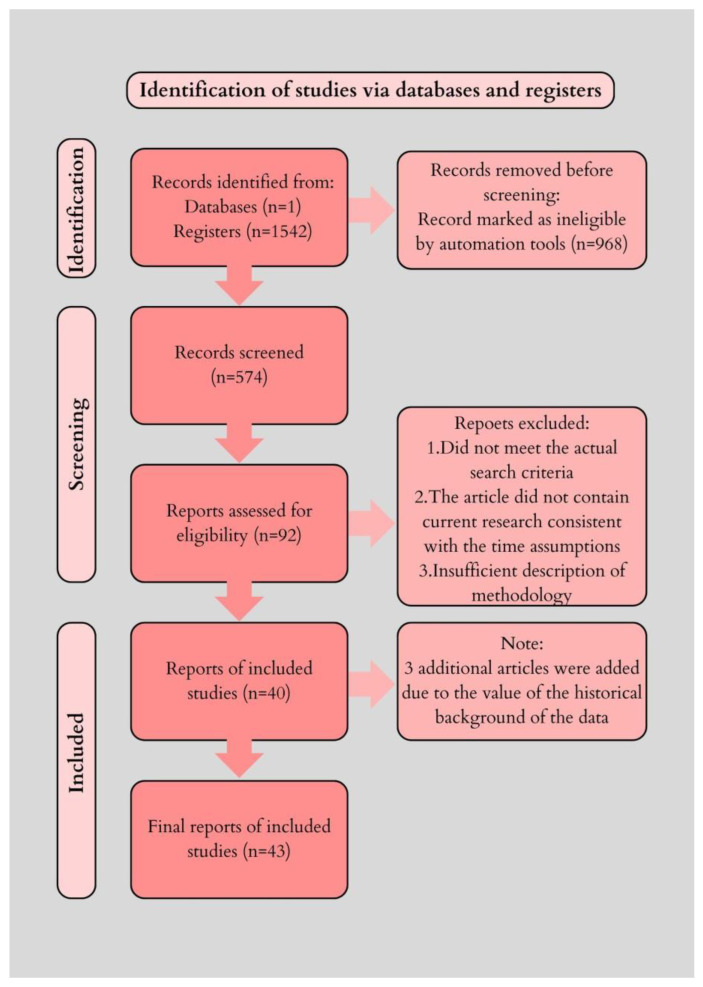
PRISMA-like flow diagram for study selection in the review. Records identified in PubMed (*n* = 1542); Records after automatic filtering (full text since 2000) (*n* = 574); Records assessed by two independent reviewers (*n* = 92); Records included in qualitative synthesis (*n* = 40); Additional historical studies included (before 2000) (*n* = 3).

Due to the historical value, in addition to the time criteria for inclusion in the review, it was decided to include 3 articles published before the year 2000. The study selection process is illustrated in Figure 2, following the PRISMA model for transparency of inclusion and exclusion criteria. In addition, a qualitative risk-of-bias assessment was conducted for the most relevant primary studies discussed in Section 2.1 (Table 1).

## 2. Narrative Review

### 2.1. Beginnings of Electroconvulsive Therapy and Evaluation of ECT with Concomitant Hyperventilation

Interest in electroconvulsive therapy as a form of psychiatric treatment dates back to the early 1930s, when Ladislas J. Meduna injected catatonic patients with intramuscular camphor (an epileptogenic agent), which resulted in a marked improvement in their condition after a few repeated doses. This discovery inspired Italian physicians who, in 1938, under the leadership of Ugo Cerletti and Lucio Bini, conducted a study on a 39-year-old man suffering from schizophrenia. They induced ten evenly spaced seizures using 110 volts of alternating current for 0.2 s. They successfully alleviated his psychosis, allowing him to return to his wife, work, and daily life [20].

In the late 1980s and early 1990s, studies explored the effect of hyperventilation on electroconvulsive therapy (ECT) outcomes. Pande et al. (1990) examined 15 patients with depression undergoing ECT under varying hyperventilation levels [21]. Lowering pCO_2_ to around 20 mmHg significantly increased seizure duration during the first session, although this effect was not consistently observed in subsequent treatments. No major adverse effects were reported [21].

Over the years, hyperventilation has been repeatedly tested as a technique potentially enhancing the effects of ECT treatment. However, to this day, it is still not recognised as standard of care. In 1988, Räsänen performed ECT on 12 patients with depression or schizophrenia and randomly ventilated each of them with 30% oxygen and 100% oxygen [13]. The administration of 100% oxygen resulted in a 25% prolongation of the duration of seizures, compared to ventilation using 30% oxygen [13]. Similarly, Chater and Simpson, in 1988, investigated the influence of passive hyperventilation on the duration of seizures in patients undergoing ECT therapy [14]. In this study, 30 depressed patients were subjected to ECT seizures three times in a row. Transcutaneous oxyhaemoglobin saturation was monitored continuously, and EtCO_2_ was measured pre- and postictal. Hyperventilation resulted in a significant prolongation of seizure duration and reduced postictal EtCO_2_ [14]. Similar conclusions were reached by Sawayama et al. in 2008, Di Pauli et al. in 2009, and Mayur et al. in 2010 [15,16,17]. However, Haeck et al. (2011) showed that hyperventilation also allowed for a lower electrical charge by increasing the energy of the seizure during ECT [18]. The study involved 114 patients diagnosed with depression or psychosis, undergoing ECT for the first time, with a laryngeal mask used for airway protection and mechanical ventilation [18].

**Figure 3:**
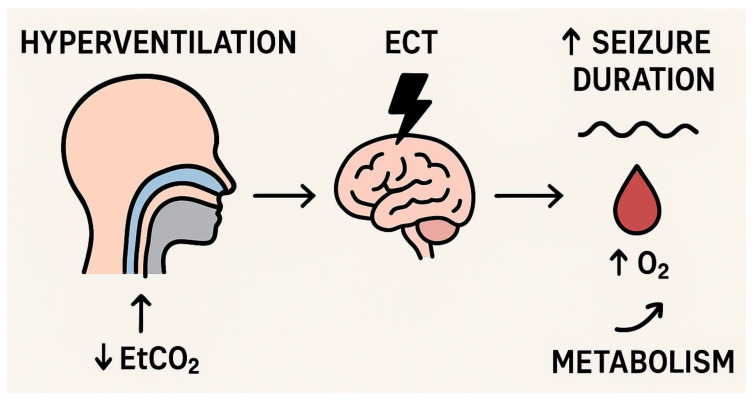
Mechanism of the influence of hyperventilation on electroconvulsive therapy (ECT). Hyperventilation leads to a reduction in end-tidal carbon dioxide (↓ EtCO_2_), resulting in increased arterial oxygenation (↑ O_2_) and modulation of metabolic processes in the central nervous system. These changes enhance brain susceptibility to electrical stimulation, which may prolong seizure duration and potentially improve the efficacy of electroconvulsive therapy.

Hyperventilation manoeuvres are controversial due to the effects of subsequent hypocapnia. Hypocapnia means an abnormally low level of carbon dioxide concentration in the blood. Carbon dioxide is a metabolic byproduct excreted from the body through respiration. Hyperventilation, translating to overly increased tidal volumes, leads to excessive exhalation of CO_2_. Among the effects of hypocapnia are transient metabolic alkalosis and vasoconstriction, which could potentially affect cerebral blood flow and the duration of seizures and evoke cognitive impairment. Conversely, increased blood flow to the brain observed during a seizure may have a neuroprotective effect [17]. Patients burdened with heart disease or in a state of hypovolemia are at an increased risk of myocardial ischemia during hyperventilation. Hyperventilation is also contraindicated in the case of pregnant patients and those suffering from chronic obstructive pulmonary disease (COPD), due to the increased risk of possible complications. However, through ECT sessions, hyperventilation is usually elicited briefly and is considered safe. Additionally, hyperventilation may reduce some of the side effects of ECT, such as post-therapy headaches and post-seizure delirium. Research into optimal hyperventilation protocols and monitoring is needed to reduce the cognitive side effects associated with higher-intensity ECT stimuli. Measuring EtCO_2_ may be a practical, minimally invasive method for assessing and monitoring CO_2_ levels during ECT sessions, especially when using a face mask during hyperventilation [12]. These mechanisms are summarised schematically in Figure 3.

The above-mentioned mechanisms were described in a study by Aida de Arriba-Arnau and Antonio Dalmau in 2017 [19]. Moreover, the authors indicated that the hyperventilation-based protocol yielded promising results in optimising the ECT technique. The strategy is based on intra-procedural carbon dioxide monitoring, enabling the adjustment of oxygen concentration administered to the patient, depending on the ECT session phase, to decrease the risk of consequent metabolic disorders. Should the method of hyperventilation in patients undergoing ECT be standardised, this strategy could be used not only for more severe and complex cases but also in routine clinical practice, unless other medical contraindications are discovered. Another study evaluated the effects of manual hyperventilation manoeuvres using face masks, monitored by capnography, and applied in a systematic and repeatable manner called reflex hyperventilation (rHV). The study aimed primarily to analyse changes in ventilation parameters during ECT sessions in which rHV manoeuvres were used, assuming that a reduction in EtCO_2_ values would be observed. rHV-induced hypocapnia was correlated with longer seizure duration but did not correlate with other seizure quality characteristics. In turn, oxygen saturation (O_2_) correlated with some parameters of seizure quality but did not affect seizure duration. It is feasible that O_2_ and CO_2_ interact synergistically, affecting seizure quality [18,19].

Preliminary clinical recommendations suggest considering hyperventilation in patients who have treatment-resistant depression, provided that contraindications such as COPD or pregnancy do not exist. EtCO_2_ and oxygen saturation should be continuously monitored. This ensures safety. However, formal guidelines have not yet been established.

### 2.2. Innovations and New Research Directions

Scientific evidence suggests that in patients managed with induced hyperventilation, seizures last longer, and the time needed to regain full consciousness is shorter. Hyperventilation affects brain tissue oxygenation, based on patient observation at different time points during therapy sessions. On the contrary, some studies show no significant differences in brain tissue oxygenation between patients managed with hyperventilation and those maintained under normocapnia. However, there are still no definitive clinical guidelines regarding the use and optimal protocol of hyperventilation during electroconvulsive therapy [19,22].

Regarding innovations, this somewhat controversial therapy management, combining traditional ECT with concomitant hyperventilation, may potentially evolve under the influence of several substantial factors. Current research on the effectiveness of ECT with hyperventilation leaves many questions regarding its benefits and probable side effects. By minimising the risk of side effects even further, the safety profile of the method increases, thus paving the way for more frequent use in daily clinical practice [23,24]. Such a goal may be achieved by continuous monitoring of end-tidal carbon dioxide, interictal and peri-ictal electroencephalogram parameters, and cardiovascular reactions, as well as by monitoring the quality of the ventilation via oxygen face masks, modifying treatment protocols, and revising the routinely administered drugs [25,26].

Moreover, the development of medical technologies, i.e., progress in electroconvulsive therapy, may enhance the efficacy of the procedure itself. Modern devices can enable more accurate and personalised delivery of electrical impulses [27]. Magnetic resonance spectroscopic imaging (MRSI) is one of the tools that uses phase coding to create spatial maps of magnetic resonance (MR) signals from various chemicals in organisms. Unlike the standard MRI method, which generates images based on signals from water molecules, MRSI allows for obtaining MR signals from various metabolites, such as N-acetylaspartate (NAA) or choline-containing compounds. This technique is beneficial in fields such as neurology, psychiatry, and psychology because it allows real-time evaluation of metabolite concentration changes. Confirming that the NAA concentration remains within normal range after ECT suggests the absence of significant neuronal damage, whereas a decrease in the NAA concentration may indicate the occurrence of such neuronal injury. However, the decline in the NAA concentration may reflect reversible changes in neuronal metabolism rather than permanent changes in neuron count or neuronal density. Notably, most MRSI studies remain in the preliminary phase and are characterised by varying electroconvulsive therapy protocols, heterogeneous patient study groups, and different brain regions analysed [28,29,30]. Another innovative idea to confirm the effectiveness of ECT is the assessment of neurotrophins, specifically the brain-derived neurotrophic factor (BDNF), which plays an essential role in the processes of endurance, plasticity, and migration of neurons. It was hypothesised that depressive states are associated with reduced BDNF expression in limbic areas, which may contribute to the atrophy of these structures. This hypothesis also suggests that antidepressant treatment may induce neurotrophic effects that reverse neuronal cell loss, leading to therapeutic effects. Studies have confirmed that the BDNF concentration is reduced in mood disorders, and an increase in BDNF concentration is associated with clinical improvement in depression. Therefore, an elevation of its serum concentration after ECT may further confirm that ECT therapy can provoke neurogenesis in the hippocampus and other limbic areas, affecting its therapeutic outcomes [28,31].

A personalised therapy attitude is also vital in ECT therapy. Understanding patients’ specific needs and tailoring the hyperventilation technique may minimise side effects and increase treatment effectiveness. An individualised approach to therapy may contribute to better outcomes [32]. Such an approach to maximising ECT’s effectiveness and minimising cognitive side effects involves treating patients according to their seizure threshold. Additionally, the method of anaesthesia (including the choice of a specific anaesthetic agent and its dose), the parameters of the ECT device used (such as current amplitude and frequency), and parallel lithium treatment may modify the seizure threshold [33]. One of the biggest challenges after ECT is maintaining remission, which is troublesome, as depressive episodes tend to relapse within a few weeks. Various strategies, such as pharmacotherapy continuation after ECT, have been investigated, but it has been noted that the effectiveness of these approaches may be limited, especially in drug-resistant cases. The effectiveness of systematically repeated ECT sessions as a way to prolong remission has also been studied, but currently, there are no decisive guidelines on this issue. The up-to-date approach is based on aligning patients’ symptoms to the ECT sessions’ schedule. The Symptom-Titrated, Algorithm-Based Longitudinal ECT (STABLE) algorithm aims to provide a more personalised approach to maintaining remission after ECT and is yet to be widely tested in randomised controlled trials [34].

With the development of new strategies for mental disorders treatment, such as pharmacotherapy, psychoeducational therapies, and complementary therapies, the use of ECT with protocolised hyperventilation may decline. The future of this therapy may depend on popularisation and accessibility from other, more advanced and less invasive treatment options [12,19]. The strongest competitor is Transcranial Magnetic Stimulation (TMS), which is already gaining popularity in clinical practice. However, while having few side effects, it is considered less effective than ECT [35]. In contrast, Magnetic Seizure Therapy (MST) uses magnetic pulses to evoke seizures. There are no large-scale studies yet, but preliminary studies indicate faster recovery and fewer side effects or cognitive impairments [36]. Another method of treating mental disorders is vagus nerve stimulation used, for instance, in the treatment of severe and resistant epilepsy and depression. However, current evidence regarding the safety and effectiveness of this method is insufficient, and further large-scale testing is encouraged [37]. In addition, a method involving transcranial direct current stimulation, targeted at cortical areas via electrodes on the scalp, is also being investigated. Specific reports or guidelines are yet to be made available [38]. Deep brain stimulation (DBS), widely utilised for the management of numerous neurological disorders, has been shown to suppress various symptoms by stimulating sites in the anteromedial hypothalamic nucleus and ventral capsule or ventral striatum, but it has not yet been found superior to currently used ECT [27,39,40].

There is an ongoing issue of lack of social acceptance for ECT. This remains a challenge due to the many controversies surrounding this treatment method. Social and educational campaigns and access to reliable sources of information can help society members understand the basics and benefits of ECT. Such campaigns should be promoted in schools and medical universities to spread knowledge and avoid misconceptions. Moreover, physicians and other medical workers should be willing to talk openly with patients and their families about ECT and explain its benefits and possible risks [40]. A plethora of evidence-based scientific studies already exist regarding the effectiveness of ECT and its risk–benefit ratio, proving the value of this technique [41,42,43].

Although many centres already utilise protocolised hyperventilation based on published research results, there is still room for improvement, as it is not routinely implemented in all ECT cases, despite increasing patient safety and effectiveness of the procedure [42,44].

Future studies on hyperventilation in ECT should aim to plan for much larger multicentre randomised controlled trials instead of smaller single-centre observations [17,18,19,22]. These studies should evaluate not only seizure duration and stimulus efficiency but also long-term remission rates [4,34], the effectiveness of strategies in preventing relapse [4], and the resulting cognitive outcomes [28]. Researchers must investigate this systematically, since oxygenation and carbon dioxide levels might interact [12,19]. Including differences between manual and mechanical hyperventilation techniques [25]. Researchers should also consider integrating continuous EtCO_2_ together with O_2_ monitoring as part of standard ECT protocols [23]. They should also assess the role of hyperventilation in personalised therapy approaches [32,33]. Such studies may provide the evidence base necessary in order to establish preliminary clinical guidelines that ensure the safe and effective implementation of hyperventilation during ECT.

## 3. Conclusions

Hyperventilation throughout electroconvulsive therapy modifies oxygen, along with carbon dioxide levels, because they synergistically influence seizure duration and seizure quality. Seizure activity is prolonged by the reduced amount of CO_2_, and the risk of hypoxia may be lowered by an increased level of oxygen delivery. These mechanisms might improve ECT’s full efficacy. Therefore, we must monitor CO_2_ concentration. Hyperventilation can occur during monitoring. Table 2 summarizes the studies that investigated hyperventilation in ECT. Approaching each patient individually remains fundamental, as protocolised hyperventilation may further increase treatment effectiveness. Current ECT guidelines address various aspects of the procedure, including electrical dose, anaesthesia management, and patient monitoring. Hyperventilation, however, is not specifically addressed within these guidelines. Future studies aimed at improving medical practice should focus on developing standardised induced-hyperventilation protocols and validating them so that patients achieve faster, more favourable outcomes and enhanced therapeutic efficacy.

## Figures and Tables

**Table 1:** The qualitative risk-of-bias assessment.

Author, Year	Study Design	Sample Size	Centre Type	Main Limitations	Risk of Bias
Räsänen, 1988 [13]	Prospective, controlled	12	Single-centre	Very small sample, old ECT protocols	High
Chater & Simpson, 1988 [14]	Prospective	30	Single-centre	No randomisation, limited monitoring technology	Moderate
Sawayama, 2008 [15]	Prospective	40	Single-centre	Small sample, heterogeneous patient group	Moderate
Di Pauli, 2009 [16]	Prospective	36	Single-centre	Small sample, non-randomised	Moderate
Mayur, 2010 [17]	Prospective	32	Single-centre	Small sample, limited seizure quality assessment	Moderate
Haeck, 2011 [18]	Observational	114	Single-centre	Non-randomised, heterogeneous psychiatric diagnoses	Moderate
Arriba-Arnau, 2017 [19]	Protocolised intervention	52	Multi-centre	Modest sample size, limited follow-up	Low/Moderate

**Table 2:** Summary of studies on hyperventilation in ECT.

Author, Year	Sample Size	Population	Ventilation Protocol	Target EtCO_2_ (mmHg)	O_2_ Saturation (%)	Change in Seizure Duration	Key Findings
Pande et al., 1990 [21]	15	Patients with depression	Varying hyperventilation levels during ECT	~20	Not reported	Increased seizure duration during first session	Lowering pCO_2_ increased seizure duration in the first session without major adverse effects
Chater & Simpson, 1988 [14]	30	Patients with depression undergoing ECT	Passive hyperventilation, EtCO_2_ measured pre- and postictal	25–30 (reduced postictal)	Continuously monitored	Significant prolongation	Hyperventilation significantly prolonged seizure duration and reduced EtCO_2_.
Sawayama et al., 2008 [15]	38	Patients undergoing ECT	Hyperventilation (manual ventilation with 100% O_2_)	<30	99–100	Prolonged	Hyperventilation prolonged seizure duration compared to normoventilation.
Di Pauli et al., 2009 [16]	42	Patients undergoing ECT	Controlled hyperventilation	<30	Not specified	Prolonged	Hyperventilation increased seizure duration significantly.
Mayur, 2010 [17]	40	Patients undergoing ECT	Hyperventilation vs. normoventilation	25–30	Not reported	Prolonged	Hyperventilation improved seizure efficiency and duration.
Haeck et al., 2011 [18]	114	Patients with depression or psychosis (first-time ECT)	Controlled hyperventilation with laryngeal mask and mechanical ventilation	25–30	Not reported	Prolonged; lower electrical charge required	Hyperventilation increased seizure energy and reduced charge requirement.
de Arriba-Arnau & Dalmau, 2017 [19]	51	Patients undergoing ECT	Protocolised hyperventilation with EtCO_2_ and O_2_ monitoring	25–30	Correlated with seizure quality	Prolonged	rHV prolonged seizure duration; O_2_ correlated with seizure quality, not duration.
Gündoğdu, 2019 [18]	62	Patients undergoing ECT	Hyperventilation vs. normoventilation	25–30	98–100	Prolonged	Hyperventilation prolonged seizure length and improved oxygenation.

## Data Availability

Not applicable.

## Abbreviations

BDNF	brain-derived neurotrophic factor
CNS	central nervous system
COPD	chronic obstructive pulmonary disease
DBS	deep brain stimulation
ECT	electroconvulsive therapy
EtCO_2_	end-tidal CO_2_
MR	magnetic resonance
MRSI	spectroscopic imaging
MST	Magnetic Seizure Therapy
NAA	N-acetylaspartate
O_2_	oxygen saturation
rHV	reflex hyperventilation
STABLE	Symptom-Titrated, Algorithm-Based Longitudinal ECT
TMS	Transcranial Magnetic Stimulation
